# Effects of Andrographolide on Mouse Intestinal Microflora Based on High-Throughput Sequence Analysis

**DOI:** 10.3389/fvets.2021.702885

**Published:** 2021-08-18

**Authors:** Haigang Wu, Xian Wu, Li Huang, Chongmei Ruan, Jinni Liu, Xiaoqing Chen, Jicheng Liu, Houqing Luo

**Affiliations:** ^1^College of Animal Science and Veterinary Medicine, Xinyang Agriculture and Forestry University, Xinyang, China; ^2^College of Animal Science, Wenzhou Vocational College of Science and Technology, Wenzhou, China

**Keywords:** high-throughput sequencing, gut microbiota, andrographolide, antibiotics, 16S rRNA

## Abstract

The intestinal flora is a micro-ecosystem that is closely linked to the overall health of the host. We examined the diversity and abundance of intestinal microorganisms in mice following the administration of andrographolide, a component of the Chinese medical herb *Andrographis paniculata*. Administration of andrographolide produces multiple beneficial effects including anti-inflammatory, antiviral and antibacterial effects but whether it directly influences the gut microbiota is not known. This study investigated whether the oral administration of andrographolide influences the intestinal microbiota and was compared with amoxicillin treatment as a positive control and water only as a negative control. We examined 21 cecal samples and conducted a high-throughput sequencing analysis based on V3-V4 variable region of the 16S rDNA genes. We found that the diversity and abundance of mouse gut microbiota decreased in direct proportion with the amoxicillin dose whereas andrographolide administration did not affect intestinal microbial community structure. The composition of intestinal microbes following andrographolide treatment was dominated by the *Firmicutes* while *Bacteroidetes* dominated the amoxicillin treatment group compared with the negative controls. Specifically, the *f__Lachnospiraceae_ Unclassified, Lachnospiraceae_ NK4A136_group* and *Ruminococcaceae_ UCG-014* were enriched with andrographolide administration while *Bacteroides, Klebsiella* and *Escherichia-Shigella* significantly increased in the amoxicillin test groups. Amoxicillin administration altered the microbial community composition and structure by increasing the proportion of pathogenic to beneficial bacteria whereas andrographolide administration led to increases in the proportions and abundance of beneficial bacteria. This study provides a theoretical basis for finding alternatives to antibiotics to decrease bacterial resistance and restore intestinal floral imbalances.

## Introduction

The mammalian gut microbiota is a complex micro-ecosystem and is an important contributor to host immunity, metabolism and productivity (1, 2). The activities of intestinal microbes closely parallel host growth and development and are active participates in many physiological processes (3, 4). Importantly, disease states such as obesity-related diabetes type II and other metabolic diseases (5, 6) as well as Alzheimer's, autism and other neurological diseases are tightly linked to the heath of the intestinal microbiota (7–9). Remarkably, the compounds resveratrol and berberine used in Chinese traditional medicine are effective alleviators of disease that work through interactions with the gut microbiota (10). Overall, intestinal microbes are key components of host development and health and play important roles in protecting the body from damage and maintaining health.

Andrographolide (C_20_H_30_O_5_) is the primary bioactive ingredient from the Acanthaceae family member the green chiretta (*Andrographis paniculata*) and is used in Chinese medicine. Andrographolide is a naturally occurring bicyclic diterpenoid whose other members include the bioactive compounds retinol, phytol and forskolin. Andrographolide has been shown to have anti-inflammatory (11), anti-infective (12), anti-cancer (13), anti-hyperglycemic (14) and anti-angiogenic properties (15). It also can function as an immune stimulator (16) and possesses anti-reproductive and other pharmacological effects (17). However, andrographolide is not water soluble, shows poor oral absorption with low bioavailability and is chemically unstable in body fluids (18, 19). With all these negative attributes, it is unknown whether andrographolide can alter the composition of the gut microbiota. Therefore, the objective of this study was to investigate the effect of this compound on the intestinal microbiota of mice.

## Materials and Methods

### Animals and Sample Collection

A total of seventy (*n* = 70) 28 day old healthy mice (initial weight 20 ± 2 g) were purchased from the Experimental Animal Center, Zhengzhou University (Zhengzhou, China). The proportion of males and females in each group was 1:1 to decrease the influence of sex on the microbial community. The mice self-propagated through the experimental animal center and possessed a similar genetic background. All the screened mice were subjected to the same immunization program and were determined to be free of other diseases before the experiment. Mice were raised together for 3 days and then randomly divided into seven groups, each containing ten mice including a control group (CON); high dose amoxicillin group (AMXH, 50 mg/kg), medium-dose amoxicillin group (AMXM, 20 mg/kg), low dose amoxicillin group (AMXL 5 mg/kg); high dose andrographolide group (APH, 20 mg/kg), medium-dose andrographolide group (APM, 20 mg/kg) and low dose andrographolide group (APL, 2 mg/kg). All the mice were raised in plastic cages for 14 days with a recommended standard breeding temperature (33–35°C), humidity (53–57%) and illumination time (12 h/12 h light/dark cycle). Moreover, supplies such as feed and water were supplied *ad libitum* for all groups throughout the entire experiment. Three mice were randomly selected for euthanasia in each group and the gut was excised from abdominal cavity. The separated guts were transferred to a sterilized Kraft paper and knotted with cotton rope to decrease the cross-pollution in the different intestinal segments. The contents in the intermediate sites of cecum were immediately collected and stored at −80°C until further analysis.

### DNA Extraction

DNA from the intestinal samples was extracted using a QIAamp DNA Mini Kit (Qiagen, Hilden, Germany) following the manufacturer's instructions. The quantity of DNA was measured using a NanoDrop 2000 UV spectrophotometer (Thermo Scientific, Waltham, MA, USA). Agarose gel (0.8% w/v) electrophoresis was performed to evaluate the quality and quantity of the extracted DNA.

### 16S rRNA Amplification and Sequencing

The amplification of the 16S rDNA target region (V3/V4) utilized PCR with primers (5' to 3')_338F: ACT CCT ACG GGA GGC AGC A and 806R: GGA CTA CHV GGG TWT CTA AT. DNA fragments from the gels were recovered using an AxyPrep DNA Gel Extraction Kit (Axygen-Corning, Glendale, AZ, USA). An FLx800 fluorescent microplate reader (BioTek, Winooski, VT, USA) was used for DNA quantification in conjunction with a Quant-iTPico Green dsDNA Assay Kit (Invitrogen, Waltham, MA, USA). The purified PCR products were used for constructing the sequencing library using Illumina TruSeq (Illumina, San Diego, CA, USA) following the manufacturer's specifications. Prior to sequencing, the sequencing libraries were subjected to fluorescence quantification and quality inspection. The qualified libraries were assembled, diluted and mixed in equal proportions. The final libraries were subjected to high-throughput sequencing using a MiSeq sequencing machine (GENEWIZ, Inc.).

### Bioinformatics and Statistical Analysis

The paired-end sequences achieved from high-throughput sequencing were merged into tags and the quality of raw reads were evaluated using QIIME software (Qiime1.9.1). The reads that passed the initially quality screening were assigned to the corresponding samples according to the primer and barcode information and interrogative sequences such as ambiguous bases, chimeras and mismatched primers were discarded. The obtained high-quality sequences were clustered in a operational taxonomic unit (OTU) on the basis of 97% similarity. Phylogenetic analysis and classifications were performed for the representative sequences for each OTU. The OTU richness distributions were recorded to calculate the alpha diversity. Beta diversity was performed using QIIME (Version 1.7.0) to identify similarities and differences between different samples. In addition, rarefaction curves were constructed to assess the sequencing depth. Linear discriminant analysis effect size (LEfSe) was generated to investigate the differentially abundant taxon. R (v3.0.3) and GraphPad Prism (version 8.0c) were applied to statistical analysis. The criterion of significance was conducted at *p*-values < 0.05 and the data was expressed as means ± SD.

## Results

### Quality Assessment and OTU Classification

**Figure 1:**
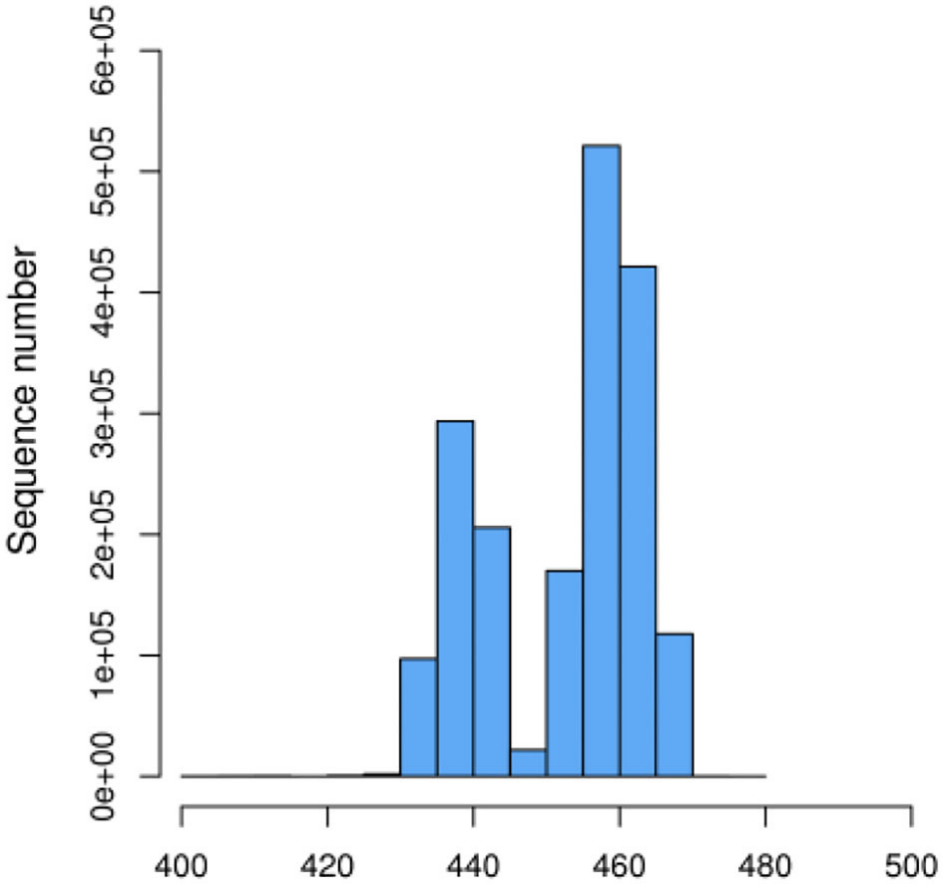
Effective sequence length distribution.

We initially performed a quality screening of our high throughput sequencing data to eliminate erroneous and questionable sequences to verify sequence reliability. The data was optimized and produced 1,756,513 high-quality reads with an average of 56,662 reads (range 53,560–106,150) per sample (Table 1) and a sequence length of 443–460 bp (Figure 1).

**Table 1:** The sequence information of each sample.

**Sample**	**Raw_reads**	**Qualified_Reads**	**Efective (%)**
APL1	123,587	106,150	86.28
APL2	95,524	87,432	85.89
APL3	104,216	92,830	89.07
APM1	101,234	69,924	80.53
APM2	100,140	91,701	87.65
APM3	109,961	96,342	88.05
APH1	59,824	53,560	89.34
APH2	101,820	93,394	82.23
APH3	117,461	101,512	86.42
AMXL1	117,503	100,193	85.26
AMXL2	81,257	62,843	77.33
AMXL3	88,733	74,240	83.66
AMXM1	115,031	91,710	79.72
AMXM2	99,838	84,482	84.61
AMXM3	108,419	88,871	81.96
AMXH1	99,561	83,134	83.50
AMXH2	75,849	62,213	82.02
AMXH3	107,177	82,495	76.97
CON1	94,668	72,573	76.66
CON2	106,411	93,561	87.92
CON3	86,873	67,353	77.53

**Figure 2:**
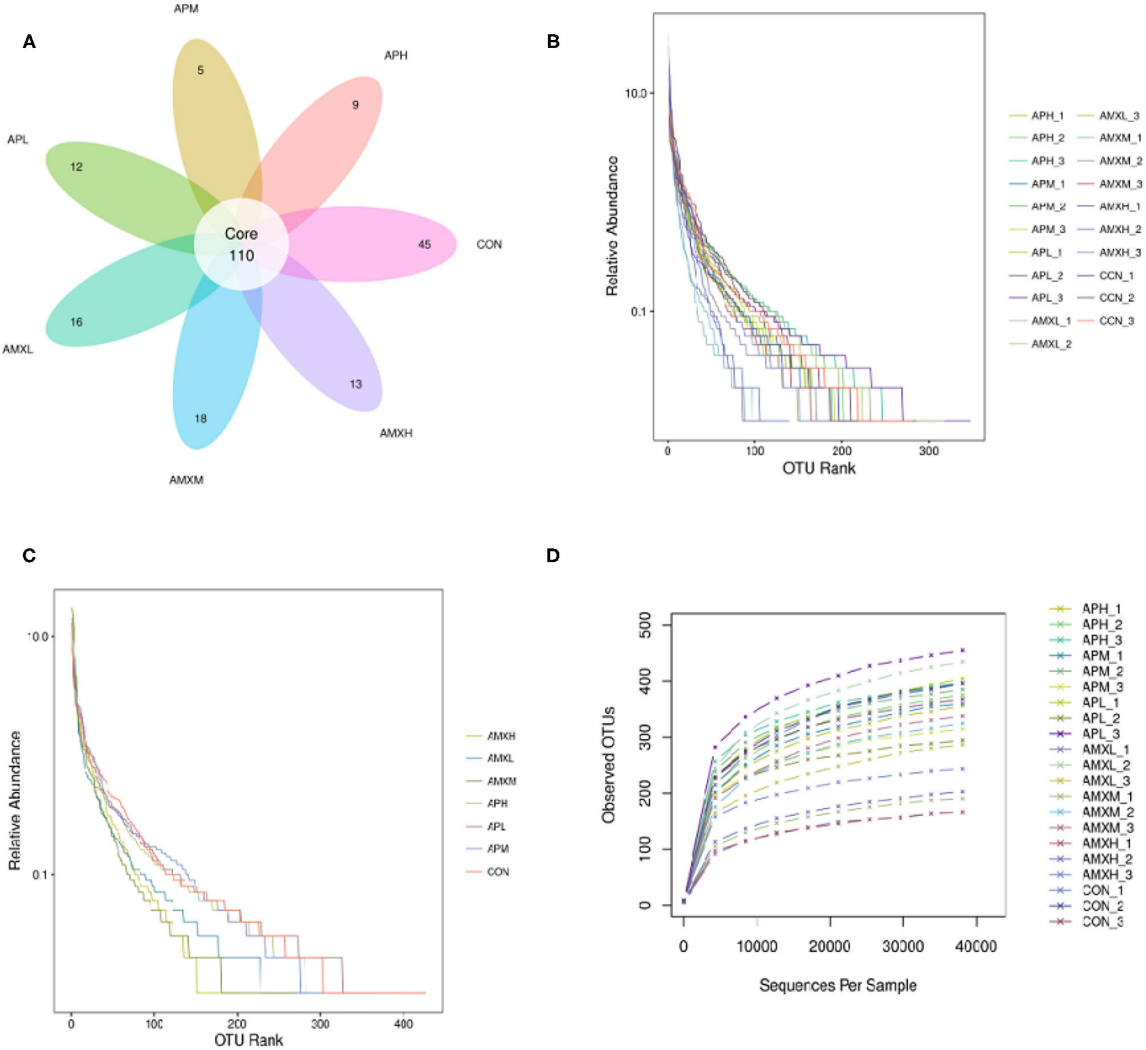
Venn diagrams and feasibility analysis **(A–D)**.

The qualified reads comprised 110 OTUs on the basis of 97% nucleotide-sequence identity (Figure 2A). The rarefaction and rank abundance curves per sample were relatively flat and displayed a tendency to saturate suggesting that the depth and evenness of the sequences meets the requirements for sequencing and analysis (Figures 2B–D).

### Analysis of Microbial Community Diversity

**Figure 3:**
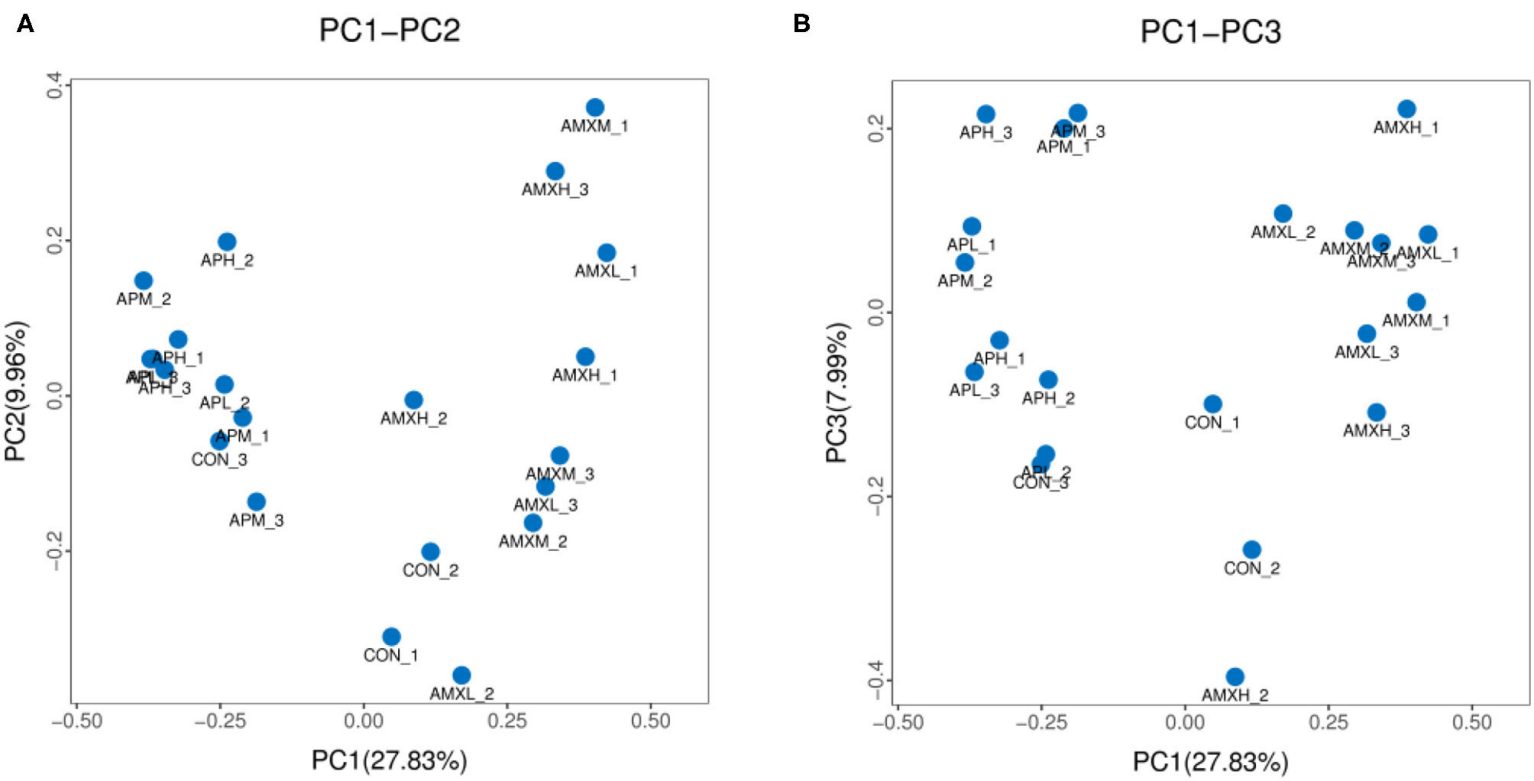
PCoA analysis of intestinal microbial community in different groups. **(A,B)** represent PCoA map on the basis of unweighted and weighted uniFrac distance, respectively.

We assessed the alpha diversity of our gut microbiota samples and the Good's coverage estimates varied from 99.8 to 99.9% for all of the samples indicating excellent coverage (Table 2). The Chao1 indices for the experimental groups AMXL, AMXM, and AMXH were 354.61, 323.79, and 250.54 and the corresponding ACE indices were 355.85, 327.99, and 249.51, respectively. Moreover, the averages of Shannon indices for these 3 groups were 4.67, 4.44, and 4.27, respectively. The Chao1, ACE, Shannon, and Simpson indices for these groups treated with AMX displayed a gradual downward trend as the drug concentration was increased indicating that AMX reduced the abundance and diversity of the intestinal microbial community. Remarkably, the three diversity indices (ACE, Chao1, and Shannon) of the control group were higher than those of the AMX treatment groups. In contrast, significant differences in gut microbiota abundance and diversity were only observed between the control and AMXH group. The average for the Chao1 index in the andrographolide treatment groups (APH, APM and APL) ranged from 412.64 to 463.67 while the ACE index ranged from 405.47 to 439.24. Moreover, the average values for the Shannon index in the andrographolide treatment group ranged from 5.50 to 5.70. Interestingly, the Chao1 and ACE index for APH was higher compared with APL and APM and the 4 diversity indices for APM were lower when compared to APL, APM and CON. However, differences between the 4 groups were not significant (*P* > 0.05). The α-diversity indices revealed no significant difference in the diversity and richness of gut microbiota between the andrographolide and negative control groups. Both the weighted and the unweighted PCoA plots revealed that the samples in each group were clustered separately indicating differences in the gut microbiota for the samples (Figure 3).

**Table 2:** Microbial diversity index analysis.

**Sample**	**Chao1**	**ACE**	**Shannon**	**Simpson**
APL	438.56 ± 80.47	437.00 ± 81.78	5.57 ± 0.31	0.94 ± 0.019
APM	412.64 ± 20.38	405.47 ± 25.64	5.63 ± 0.34	0.95 ± 0.016
APH	463.67 ± 22.49	439.24 ± 8.56	5.50 ± 0.43	0.94 ± 0.020
AMXL	354.61 ± 125.15	355.81 ± 121.13	4.67 ± 0.92	0.89 ± 0.058^*^
AMXM	323.80 ± 76.82	327.99 ± 78.01	4.44 ± 0.66^*^	0.88 ± 0.041^*^
AMXH	250.54 ± 38.87^*^	249.51 ± 33.95^*^	4.27 ± 0.24^*^	0.88 ± 0.014^*^
CON	439.23 ± 14.67	444.46 ± 19.06	5.75 ± 0.28	0.96 ± 0.0070

*Chao1, ACE, Shannon, and Simpsonwere calculated to assess the alpha diversity of intestinal microbial community. The data are expressed as the mean ± SD*.

* *P < 0.05*.

### Bacterial Community Composition in Groups

**Figure 4:**
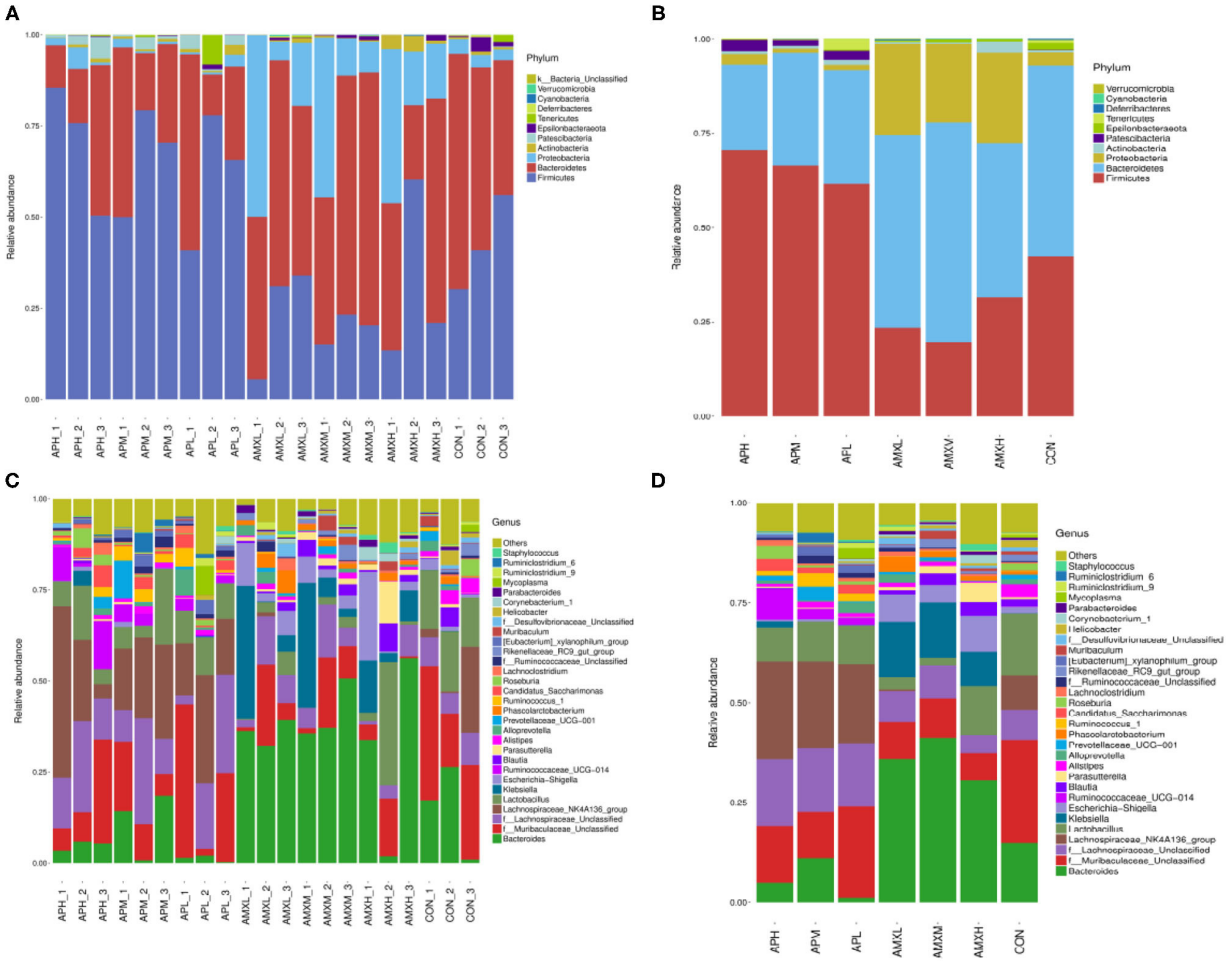
The relative abundance of the gut microbiota at the phylum **(A,B)** and genus **(C,D)** levels.

We obtained that were comprised of 11 phyla, 17 classes, 28 orders, 55 families, 157 genera and 168 species. The *Firmicutes, Bacteroidetes* and *Proteobacteria* were the 3 dominant phyla for all samples and represented 45.09, 40.46, and 11.12%, of the totals, respectively. These phyla constituted the core of the microbiota and accounted for 96.67% of the taxonomic groups identified. The andrographolide treatment groups were primarily composed of *Firmicutes* (66.21%) and *Bacteroidetes* (27.51%) and *Proteobacteria* (1.83%). The *Firmicutes, Bacteroidetes* and *Proteobacteria* content for the AMX treatment groups accounted for 24.88, 50.07, and 22.97% of the total, respectively. The dominant phyla for the negative control group were *Bacteroidetes, Firmicutes*, and *Proteobacteria* and were accounted for by 50.55, 40.40, and 3.46% of the totals, respectively (Figure 4).

The genus *Lachnospiraceae_NK4A136* comprised 24.64 and 21.65% of the overall bacterial composition in the APH and APM groups while *f_Lachnospiraceae_Unclassified* were represented at 16.74 and 15.80%, respectively. The levels of these two genera in the APH and APM groups were significantly higher than the negative control and AMX treatment groups (*P* < 0.05). In the APL test group, the most numerous genera were *f_Muribaculaceae Unclassified* and *Lachnospiraceae_NK4A136* at 22.79 and 19.77% of the overall bacterial composition, respectively. The levels of the latter genra were significantly higher than the negative control and AMX groups (*P* < 0.05).

**Figure 5:**
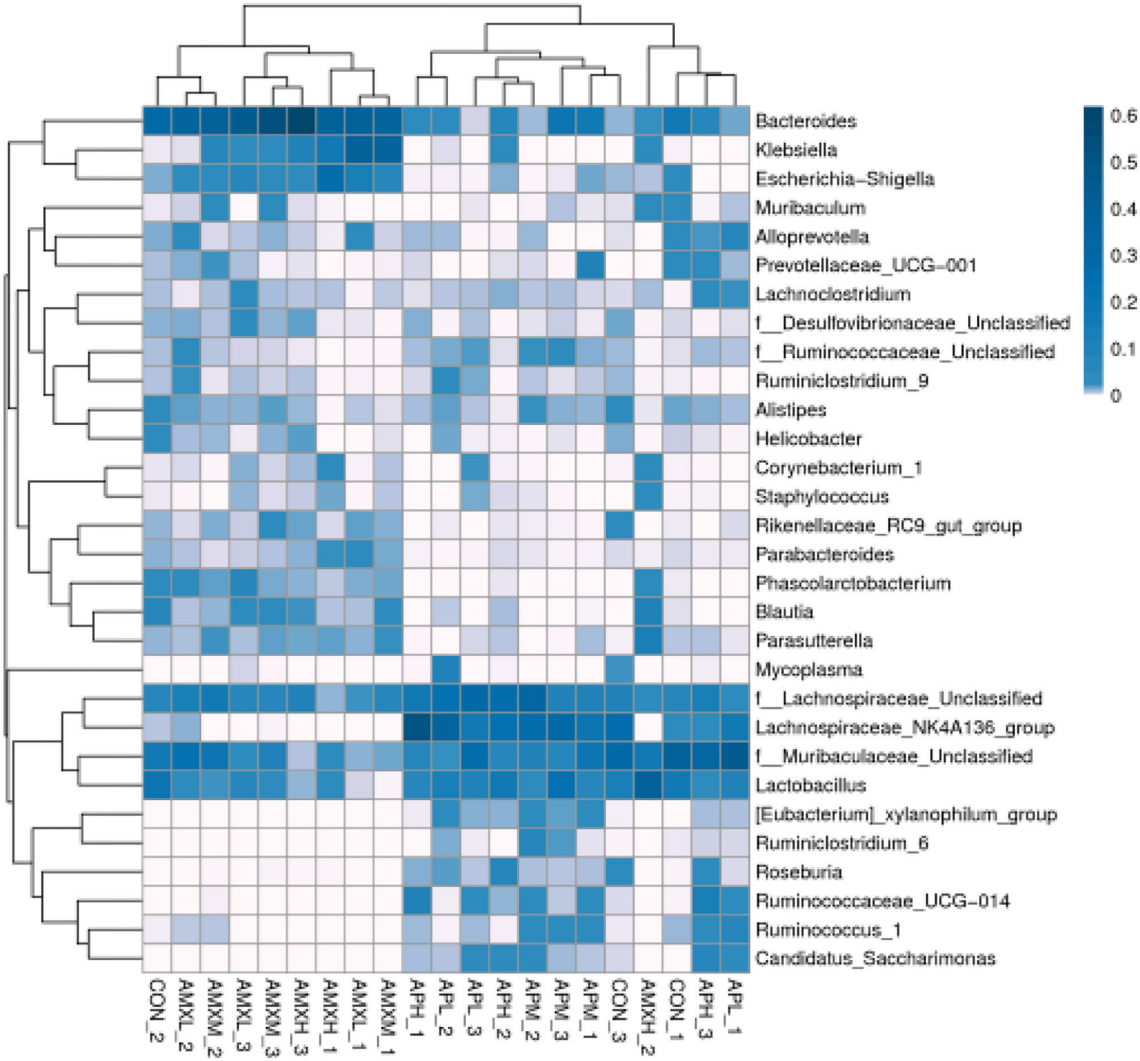
Heatmap of the relative richness of bacterial communities at the genus level. Each color-block in the heatmap represents the relative richness of a bacterial genus in a sample.

The most abundant genera for the AMX treatment groups were *Bacteroides* at 35.87, 41.11, and 30.26% for AMXL, AMXM, and AMXH, respectively. *Klebsiella* was second most abundant for these groups at 13.74, 13.94, and 8.45%, respectively. These levels for *Bacteroides* and *Klebsiella* were significantly higher than for the negative control and andrographolide treatment groups (Figure 5).

We performed a metastatic analysis to identify significantly different taxa between the different test groups (Tables 3–11). Our results revealed that at the genus level, the abundance of *Candidatus_Saccharimonas* increased while the *Lactobacillus, Escherichia-Shigella* and *Alistipes* genera decreased significantly in the APL group; The abundance of *Ruminococcaceae* also increased significantly while *Alistipes* decreased significantly in the APM and APH groups, respectively. The abundance of *Bacteroides* in the AMXL and AMXM groups was higher than negative controls while Lactobacillus levels were lower than negative controls. In the latter, *Corynebacterium_1* and *Klebsiella* increased significantly while *f_Muribaculaceae_Unclassified* and *Alipis* decreased significantly in the AMXH group. Compared with the AMX test groups, *Lachnospiraceae_NK4A136p, Lactobacillus* and *Candidatus_Saccharimonas* abundance in the APL group were significantly higher than for the AMXL group while *Bacteroides, Escherichia-Shigella*, and *Phoscolarctobacterium* in the APL group were significantly lower than for the AMXL group. Compared with the AMXM group, the abundance of *Lachnospiraceae_NK4A136, Ruminococcus_1, f_Ruminococcaceae_Unclassified* and *[Eubacterium]_xylanophilum_group* increased significantly and was accompanied by a significant decrease in the abundance of *Bacteroides Escherichia-Shigella, Blautia* and *Parasutterella* for the APM group. The AMXH and APH groups displayed a significant increase in the abundance of *f_Lachnospiraceae_Unclassified, Lachnospiraceae NK4A136_group, Ruminococcaceae_UCG-014, Candidatus_Saccharimonas and Roseburia* as well as a significant decrease in the abundance of *Phascolarctobacterium* and *Corynebacterium_1*.

**Table 3:** Based on Metastats analysis for flora differences between APL and Con groups under the genus.

**Taxon (genus)**	**Group1_mean**	**Group1_mean**	***P*-Value**
	**APL**	**Con**	
Lactobacillus	0.096655015	0.154013706	≤0.001
Escherichia-Shigella	0.00046694	0.016875919	0.030695652
Alistipes	0.008404823	0.031416406	0.020913043
Candidatus_Saccharimonas	0.022779083	0.000833917	0.047173913

**Table 4:** Based on Metastats analysis for flora differences between APM and Con groups under the genus.

**Taxon (genus)**	**Group1_mean**	**Group1_mean**	***P*-Value**
	**APM**	**Con**	
Ruminococcaceae_UCG-014	0.028215847	0.00100067	0.046
Alistipes	0.013875292	0.031416406	0.045
Ruminococcus_1	0.032751995	0.003668828	0.0037
f__Ruminococcaceae_Unclassified	0.020245024	0.004902668	0.032

**Table 5:** Based on Metastats analysis for flora differences between APHL and Con groups under the genus.

**Taxon (genus)**	**Group1_mean**	**Group1_mean**	***P*-Value**
	**APH**	**Con**	
f__Lachnospiraceae_Unclassified	0.167429058	0.074875512	0.0418
Ruminococcaceae_UCG-014	0.078367449	0.00100067	0.049
Alistipes	0.006536101	0.031416406	0.02255

**Table 6:** Based on Metastats analysis for flora differences between AMXL and Con groups under the genus.

**Taxon (genus)**	**Group1_mean**	**Group1_mean**	***P*-Value**
	**AMXL**	**Con**	
Bacteroides	0.359057169	0.14836296	0.0186
Lactobacillus	0.031633386	0.154013706	0.0049
Alistipes	0.0097429	0.031416406	0.0282
Phascolarctobacterium	0.03850549	0.007335664	0.053

**Table 7:** Based on Metastats analysis for flora differences between AMXM and Con groups under the genus.

**Taxon (genus)**	**Group1_mean**	**Group1_mean**	***P*-Value**
	**AMXM**	**Con**	
Bacteroides	0.4113589	0.14836296	0.025473684
Parasutterella	0.0188766	0.004635244	0.010263158
Lactobacillus	0.016674292	0.154013706	≤0.001

**Table 8:** Based on Metastats analysis for flora differences between AMXH and Con groups under the genus.

**Taxon (genus)**	**Group1_mean**	**Group1_mean**	***P*-Value**
	**AMXH**	**Con**	
f__Muribaculaceae_Unclassified	0.06852468	0.258282341	0.048842105
Klebsiella	0.084603602	0.000633583	0.043
Alistipes	0.00333408	0.031416406	0.010368421
Corynebacterium_1	0.021109588	0.000867054	0.030894737

**Table 9:** Based on Metastats analysis for flora differences between APL and AMXLon groups under the genus.

**Taxon (genus)**	**Group1_mean**	**Group1_mean**	***P*-Value**
	**APL**	**AMXL**	
Bacteroides	0.012540377	0.359057169	≤0.001
Lachnospiraceae_NK4A136_group	0.197847269	0.003636974	0.013318182
Lactobacillus	0.096655015	0.031633386	0.017636364
Escherichia-Shigella	0.00046694	0.068888762	0.0405
Phascolarctobacterium	0	0.03850549	0.036181818
Candidatus_Saccharimonas	0.022779083	0	0.044818182

**Table 10:** Based on Metastats analysis for flora differences between APM and AMXM on groups under the genus.

**Taxon (genus)**	**Group1_mean**	**Group1_mean**	***P*-Value**
	**APM**	**AMXM**	
Bacteroides	0.111618471	0.4113589	0.024772727
Lachnospiraceae_NK4A136_group	0.216624875	0.000166763	0.004954545
Escherichia-Shigella	0.005302208	0.043795235	0.044636364
Blautia	0.000133387	0.028519585	0.039681818
Parasutterella	0.002234237	0.0188766	0.009909091
Ruminococcus_1	0.032751995	0.001433477	0.014863636
f__Ruminococcaceae_Unclassified	0.020245024	0.002400827	0.029727273
[Eubacterium]_xylanophilum_group	0.021913212	0	0.019818182

**Table 11:** Based on Metastats analysis for flora differences between APH and AMXH groups under the genus.

**Taxon (genus)**	**Group1_mean**	**Group1_mean**	***P*-Value**
	**APH**	**AMXH**	
f__Lachnospiraceae_Unclassified	0.167429058	0.044311357	0.0147
Lachnospiraceae_NK4A136_group	0.244161782	0	0.0496
Ruminococcaceae_UCG-014	0.078367449	0	0.03025
Phascolarctobacterium	0.00053328	0.011903551	0.03505
Candidatus_Saccharimonas	0.030343342	0	0.0448
Roseburia	0.031571303	0.000166727	0.0195
Corynebacterium_1	0.00036667	0.021109588	0.0099

## Discussion

A large number of bacteria colonize the intestines of mammals and they play an indispensable role in maintaining the overall health of the host (20). The differences between the intestinal floras of different individuals are due to age, diet and health status (21). These intestinal microbes are comprised of both beneficial and harmful members. Adjusting the structure of the intestinal flora and increasing the proportion of probiotics can effectively help the host maintain a healthy state (22, 23). The active ingredients of many traditional Chinese medicines are able to reach the colon and interact with the intestinal flora. By adjusting the composition of the intestinal flora and its metabolites as well as short-chain fatty acids, the functions of the intestinal flora can be improved with beneficial effects on the host. Therefore, studying the composition and structure of intestinal microorganisms is of great significance to disease prevention and treatment (24, 25). The compound andrographolide used in the current study is a potent antibacterial. We found that increasing the levels of andrographolide did not change the alpha diversity of the intestinal flora indicating it does not interfere with the diversity and abundance of the intestinal microbial community in mice. A previous study revealed that baicalin addition to mouse diets increased the diversity index and species abundance of the intestinal flora. The number of beneficial bacteria increased while conditional pathogens such as the enterococci were reduced. Overall, andrographolide induced the emergence of a new and more structurally stable flora (26). The administration of amoxicillin reduced the abundance and diversity of the intestinal flora in mice in a dose-dependent manner in our experiments and this effect of antibiotics in general is well-documented (27, 28). Additionally, the imbalance in the intestinal microbiome caused by antibiotic administration also adversely affects host immune and endocrine systems.

We found that the proportion o*f Firmicutes* in andrographolide-treated mice was significantly higher than that of both the negative control and amoxicillin treatment groups while of the levels of *Bacteroidetes* and *Proteobacteria* were less. These results were consistent with previous high-throughput sequencing results in infants treated with antibiotics for bacterial pneumonia (29). We observed that *f_Lachnospiraceae_Unclassified, Lachnospiraceae_NK4A136_group* and *Ruminococcaceae_UCG-014* were abundant in the andrographolide treatment groups while f*_Muribaculaceae_Unclassified* and *Bacteroides* levels were the converse. Previous studies have demonstrated that *Rumenococcus* levels are negatively correlated with inflammatory markers such as C-reactive protein and IL-6. These markers are most likely the result of interactions with pathogen-related molecular patterns, bacterial metabolites, short-chain fatty acids and derivatives of trimethylamine oxide as well as bile acid metabolism. These responses affect intestinal peptide secretion and permeability while regulating the inflammatory state. The presence of *Laospirillaceae* is closely linked to butyrate metabolism and positively correlated with the presence of inflammatory cells (30, 31). Our results indicate that andrographolide can promote probiotic proliferation while inhibiting pathogenic bacteria. When compared with negative controls, the levels of *Bacteroides, Klebsiella and Escherichia-Shigella* in the amoxicillin test groups significantly increased while *f_Muribaculaceae_Unclassified* and *Lactobacillus* decreased. *Klebsiella and Escherichia-Shigella* are conditional pathogens. In addition, the presence of *Corynebacterium_1 i*n the high-dose amoxicillin group was also significantly greater than negative controls. This is another example of a reduction of the abundance and diversity of the intestinal microbial community as well as an increase in the levels of pathogenic bacteria due to antibiotic administration.

## Conclusions

This study investigated the effects of andrographolide on the intestinal microbial community of mice. The results revealed that the diversity and abundance of gut microbiota treated with AMX undergoes significant alterations that were characterized by elevated levels of harmful bacteria. Conversely, andrographolide administration significantly increased the abundance of beneficial intestinal bacteria including *f_Lachnospiraceae_Unclassified, Lachnospiraceae_NK4A136_group* and *Ruminococcaceae_UCG-014* that are indicators of the stability of the gut microbiota. These results expand the understanding of the potential benefits of andrographolide on the health of the gut microbiome.

## Data Availability Statement

The datasets presented in this study can be found in online repositories. The names of the repository/repositories and accession number(s) can be found at NCBI SRA BioProject, accession no: PRJNA743424.

## Ethics Statement

The studies were reviewed and approved by the Regulations for the Administration of Affairs Concerning Experimental Animals approved by the State Council of People's Republic of China. Written informed consent was obtained from the individual(s), and minor(s)' legal guardian/next of kin, for the publication of any potentially identifiable images or data included in this article.

## Author Contributions

HW: conceptualization, methodology, formal analysis, data curation, resources, writing-original draft, writing-review and editing, supervision, and project administration. XW: funding acquisition investigation, data curation, and writing-original draft preparation. LH: investigation, validation, data curation, formal analysis, and writing-original draft. XC: investigation, data curation, and resources. JinL: formal analysis, writing-review and editing, supervision, project administration, and funding acquisition. CR: formal analysis, resources, and validation. JicL: funding acquisition and formal analysis. HL: performed manuscript review. All authors contributed to the article and approved the submitted version.

## Conflict of Interest

The authors declare that the research was conducted in the absence of any commercial or financial relationships that could be construed as a potential conflict of interest.

## Publisher's Note

All claims expressed in this article are solely those of the authors and do not necessarily represent those of their affiliated organizations, or those of the publisher, the editors and the reviewers. Any product that may be evaluated in this article, or claim that may be made by its manufacturer, is not guaranteed or endorsed by the publisher.
